# Functional role of RRS1 in breast cancer cell proliferation

**DOI:** 10.1111/jcmm.13922

**Published:** 2018-10-15

**Authors:** Jinlian Song, Zhongliang Ma, Yanan Hua, Junting Xu, Ning Li, Chuanxia Ju, Lin Hou

**Affiliations:** ^1^ Department of Laboratory The Affiliated Women and Children's Hospital of Qingdao University Qingdao University Qingdao Shandong China; ^2^ Department of Biochemistry Medical College Qingdao University Qingdao Shandong China; ^3^ Department of Breast Surgery The Affiliated Hospital of Qingdao University Qingdao University Qingdao Shandong China; ^4^ Department of Pharmacology Qingdao University School of Pharmacy Qingdao Shandong China

**Keywords:** breast cancer, p53, proliferation, RPL11/MDM2, RRS1 gene

## Abstract

RRS1 (human regulator of ribosome synthesis 1), an essential nuclear protein involved in ribosome biogenesis, is overexpressed in some human cancers, yet its role in breast cancer remains unclear. Here, we report a functional analysis of RRS1 in breast cancer and its likely mechanism. Immunohistochemistry (IHC) and RT‐qPCR analyses indicated that RRS1 was commonly overexpressed in breast cancer tissues. The copy numbers of RRS1 were higher in tumours compared with those for normal tissues. And there was a significant correlation between copy number and mRNA expression. In addition, RRS1 overexpression was significantly correlated with lymph node metastasis and poor survival. RRS1 mRNA and protein levels were also significantly increased in a panel of human breast cancer cell lines. RRS1 knockdown inhibited proliferation and induced apoptosis and cell cycle arrest in all three cell lines. Furthermore, RRS1 knockdown suppressed the tumour formation and growth of MDA‐MB‐231 cells in nude mice. Additionally, RRS1 knockdown activated p53 and p21 in MCF‐7 cells. A marked increase in the quantity of ribosome‐free RPL11 was detected by Western blot. Moreover, co‐immunoprecipitation (CoIP) experiments showed that RRS1 knockdown activated p53 by facilitating the direct contact of MDM2 and RPL11/RPL5. Taken together, our results suggest that RRS1 may contribute to breast cancer proliferation through RPL11/MDM2‐mediated p53 activation. Therefore, RRS1 may be a promising target for breast cancer therapy.

## 1 INTRODUCTION

Breast cancer is one of the most common malignancies in females worldwide and has an increasing annual incidence; it is commonly considered to be a genetically heterogeneous disease.^1^, ^2^ Breast cancer patients usually tend to have different clinical outcome as a result of the genetic alterations in breast cancer; these alterations have been increasingly identified as the critical determinants of breast cancer initiation and progression.^3^, ^4^ Growing evidence indicates that targeted therapeutics against gene expression signatures in breast cancer have dramatically improved patient survival in recent decades.^5^ Therefore, the elucidation of these factors and their functional roles may help us to understand the progression of breast cancer better and facilitate advances in its treatment.

Ribosome biogenesis is the most well‐known function of the nucleolus, and it regulates cell growth and cell division. Several genetic diseases, such as cancer and anaemia, are commonly associated with the misregulation of ribosome biogenesis.^6^, ^7^, ^8^ Human regulator of ribosome synthesis 1 (RRS1) has been reported in yeast, where it encodes a regulatory nuclear protein consisting of 203 amino acids that is involved in ribosome biogenesis, including maturation, nuclear export and assembly.^9^, ^10^, ^11^, ^12^, ^13^ The human RRS1 homologue was subsequently identified from nucleolar extracts.^14^, ^15^, ^16^, ^17^ The mammalian RRS1 protein is localized to both the nucleolus and the endoplasmic reticulum and is involved in the endoplasmic reticulum stress response in Huntington disease.^18^ Most importantly, RRS1 expression has been recently reported in human cancers, including human colorectal and hepatocellular carcinomas.^19^, ^20^ However, the functional role of RRS1 in breast cancer and the mechanistic details of how RRS1 performs this function are currently unknown.

The 5S ribonucleoprotein particle (RNP), as a ribosomal subcomplex, consists of RPL11, RPL5 and 5S rRNA. RRS1 has been shown to regulate the nucleolar localization of the 5S RNP preribosomal complex by directly contacting the complex.^21^ And the depletion of RRS1 resulted in an increase in the nucleoplasmic accumulation of both RPL5 and RPL11. It has also been shown that the depletion of RRS1 delays rRNA processing and thus triggers 5S RNP‐mediated p53 activation and cellular senescence.^22^ Accumulating evidence indicates that all three components of 5SRNP, RPL5, RPL11 and 5srRNA in the nucleoplasm, activate p53 by binding to and inactivating MDM2 in response the nucleolar stress.^22^, ^23^, ^24^, ^25^, ^26^ RPL11 is a well‐studied participant in the p53 nucleolar stress response pathway. A recent study showed that 5S ribonucleoprotein particle (5S RNP)‐mediated p53 activation coupled perturbed ribosomal biogenesis with cell proliferation and cell cycle regulation.^21^ Furthermore, the tumour‐suppressive role of the 5S RNP‐p53 pathway through either MDM2 interaction or Hdm‐p53 checkpoint regulation has been widely studied in cancer.^26^, ^27^ We thus hypothesize that the 5S RNP‐p53 pathway plays an important role in the proliferation of breast cancer upon RRS1 knockdown.

Here, we set out to assess the functional role of RRS1 in breast cancer. We observed that the expression levels of RRS1 were higher in human breast cancer tissues than in paired non‐cancerous tissues, and high RRS1 expression levels were associated with lymph node metastasis and poor clinical outcome. Knocking down RRS1 inhibited breast cancer proliferation in vitro *and* in vivo. Our findings also provide new insights into the RPL11/MDM2/p53 pathway in the proliferation of breast cancer.

## 2 METHODS

### 2.1 Patient data

All tissue samples, including tumour samples and paired non‐cancerous (normal) tissues from the same patients, were collected from 242 female patients with operable primary breast cancer (stages I‐III) who underwent breast surgery in 2011 at the Affiliated Hospital of Qingdao University. Clinical information from patients was acquired by reviewing preoperative and perioperative medical records or by written correspondence or telephone. All patients provided informed consent, and all procedures were approved by the ethics board of the Affiliated Hospital of Qingdao University. The ages of the patients at diagnosis ranged from 29 to 70 years, with a median age of 50 years. The tissues were collected after the diagnosis was confirmed by a senior pathologist. Tumour size, the tumour, node, metastasis (TNM) stage, lymph node status, Ki67 proliferation index, oestrogen receptor (ER) status, progesterone receptor (PR) status and human epidermal growth factor receptor‐2 (HER‐2) were obtained from reviewing the medical records.

### 2.2 IHC analysis

All formalin‐fixed and paraffin‐embedded sections were analysed by IHC. Primary antibodies were used against the following targets: RRS1 (1: 1000; Abcam, Cambridgeshire, UK), p53 (1: 300; OriGene, Shanghai, China), ER (1: 300; OriGene), PR (1: 300; OriGene), HER2 (1: 300; OriGene) and Ki67 (1: 300; OriGene). The percentage of tumour cells positively stained for each antibody was semi‐quantitatively estimated. The staining intensity of RRS1 expression was scored according to the following: score 0, negative staining; score 1, weak staining; score 2, moderate staining; and score 3, strong staining; the extent of staining was classified as the percentage of positive cells: score 0, 0; score 1, 1‐25%; score 2, 26‐50%; score 3, 51‐75%; and score 4, 76‐100%. The final quantitation of staining for each sample was obtained by multiplying the two scores.^28^ RRS1 expression was graded as high expression if the score >6; if the score ≤6, the case was classified as low expression.

### 2.3 Quantification of gene copy numbers and mRNA levels

DNA from freshly frozen mammary tissues was extracted by phenol‐chloroform extraction method. Quantitative analysis of copy numbers was conducted by real‐time PCR. A qBiomarker Multicopy Reference Copy Number PCR Assay (MRef) was included on this assay. Relative gene copy numbers for each specimen were calculated as 2 × Tcopy number (tumour copy number/MRef copy number)/Ncopy number (paired non‐cancerous copy number/MRef copy number) from the same patient. RNA from freshly frozen mammary tissues, xenograft tumours and cell lines was extracted using TRIzol reagent (Invitrogen, Carlsbad, CA, USA). Quantitative real‐time PCR detection of cDNA was analysed with SYBR Green Master Mix (TransStart Tip Green qPCR SuperMix, TRAN, Beijing, China). Real‐time PCR was performed in triplicate with a CFX96 Touch Real‐Time PCR Detection System (Bio‐Rad, Hercules, CA, USA). The relative RRS1 mRNA expression was normalized to that of GAPDH.

### 2.4 Cell culture and infection

The human breast cancer cell lines MDA‐MB‐231, BT549 and MCF‐7 were cultured in high‐glucose DMEM (HyClone, Logan, UT, USA) supplemented with 8% (v/v) foetal bovine serum (Pan, Aidenbach, Germany) at 37°C. The cells were infected with retroviruses as previously described.^27^ RRS1‐targeting shRNA (shRNA1 GCTGCCTTCATTGAGTTTA) and a non‐targeting shRNA control were expressed via pSuper constitutive expression constructs (Genecard, Shanghai, China).

### 2.5 Western blot analysis

For western blotting, xenograft tumors and cell lines were lysed, and protein samples were harvested as previously described.^29^ Equal amounts of protein were resolved by SDS‐PAGE and blotted using antibodies specific to RRS1 (1:1000, Abcam), p53 (1:500, OriGene), RPL11 (1:1000, Abcam) and β‐actin (1:1000, Bioss, Beijing, China).

### 2.6 Ribosomal and non‐ribosomal fractionation

MCF‐7 cells were lysed and layered onto an 8%‐48% sucrose gradient containing 30 mmol/L Tris‐HCl (pH 7.5), 100 mmol/L NaCl and 10 mmol/L MgCl_2_ and centrifuged in a Beckman SW41 rotor for 240 minutes at 58 719 × ***g***. Fractions were collected from the top of the gradient, and the ribosomal and non‐ribosomal fractions were determined using 18S/28S rRNA as an indicator.^26^

### 2.7 CoIP

For the CoIP of endogenous proteins, MCF‐7 cells were lysed in cell lysis buffer at 4°C for 30 minutes.^26^ The resulting lysate was incubated overnight with an antibody against MDM2 (1:1000; Abcam); protein G sepharose was added, and the sample was agitated for 2 hours at 4°C. Immunoprecipitants were separated by SDS‐PAGE after washing with the same buffer and were analysed by immunoblotting with the indicated antibodies.

### 2.8 Cell proliferation, cell cycle and apoptosis assays

To measure cell proliferation, 5000 cells were plated in triplicate in a 96‐well plate, and MTT assays were performed according to the manufacturer's protocol (Sigma, Santa Clara, CA, USA). A BrdU incorporation assay was performed to detect cell proliferation according to the manufacturer's protocol (Roche, Basel, Switzerland). In addition, 3000 cells were plated in triplicate in a 96‐well plate. Cell cycle analyses were conducted with ModiFit software (BD Bioscience, New York, NY, USA) following staining with propidium iodide (50 μg/mL; Solarbio, Beijing, China) and flow cytometry (Canto II; Becton Dickinson, New York, NY, USA). Apoptosis was also evaluated by flow cytometric analyses of Annexin V‐APC binding (eBioscience, San Diego, CA, USA) along with the activity of caspase‐3 and caspase‐7 (Promega, Madison, WI, USA).

### 2.9 Xenograft tumourigenesis assay

Twenty 4‐week‐old female athymic nude mice (14‐15 g) were purchased from the Experimental Center of Beijing Vital River Laboratory Animal Technology Co., Ltd. All procedures were approved by the Animal Ethics Committee of Qingdao University. Five million MDA‐MB‐231 cells expressing RRS1‐targeting shRNA or non‐targeting shRNA were suspended in 25% Matrigel (BD Biosciences) and DMEM (BD Biosciences) and subcutaneously injected into the flank of each mouse.^30^ Tumour volume was calculated with the following formula: *v* = 0.5*xy* ^2^ (*x* = long diameter of the tumour, *y* = short diameter of the tumour, and *v* = volume). At the time of killing, the tumours were extracted, photographed and weighed. RNA and protein were extracted. Then, the mice were killed and dissected.

### 2.10 Statistical evaluation

Each experiment was performed at least three independent times. Student's *t* test was used to compare the differences between two groups. Differences in DNA copy numbers between tissue and paired noncancerous normal tissue were tested using the non‐parametric Mann‐Whitney *U* test. Pearson's correlation test was used to evaluate the associations between gene copy numbers and mRNA expression levels. The chi‐square test was used to analyse the correlation of RRS1 status with clinicopathological features. The Kaplan‐Meier method and log‐rank test was performed to determine the relationship between stratified RRS1 levels and patient survival, including overall survival (OS) and disease‐free survival (DFS). Error bars represent SD or SEM. *P* < 0.05 was considered statistical significant.

## 3 RESULTS

### 3.1 RRS1 is overexpressed in human breast cancer tissues and cell lines

**Figure 1:**
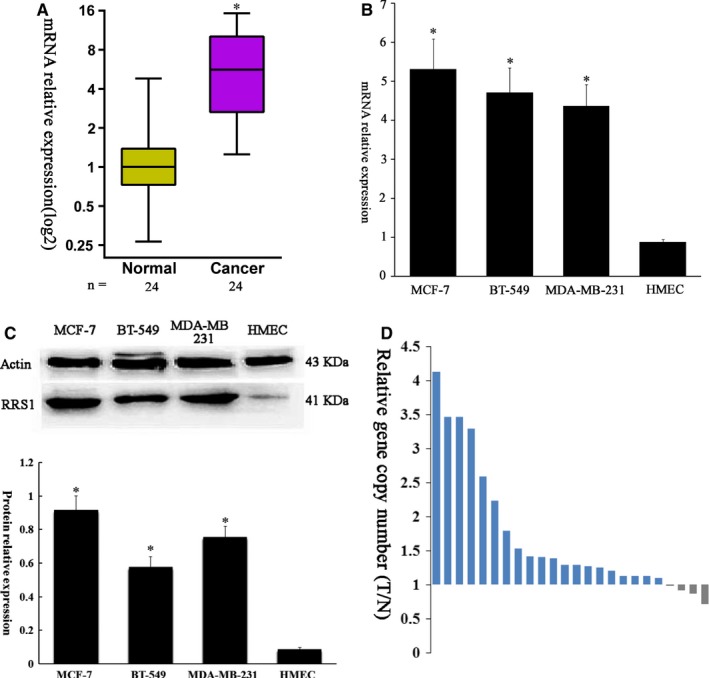
RRS1 is overexpressed in breast cancer. A, Box and whisker plot of relative RRS1 mRNA expression levels in breast cancer samples and paired non‐cancerous (normal) tissues. GAPDH was used as a reference gene. B, mRNA expression of RRS1 measured by qRT‐PCR in breast cancer cell lines and HMECs (*P < 0.01). C, Western blot analyses of whole‐cell lysates from breast cancer cells and HMECs for the proteins indicated (*P < 0.01). D, The number of copies of RRS1 gene in 24 fresh frozen breast cancer tissues and in paired non‐cancerous (normal) tissues from same patients

To evaluate RRS1 expression in human breast cancer tissues, mRNA levels in 24 freshly frozen tumours and paired non‐cancerous (normal) tissues from the same patients were measured. Our results demonstrated that RRS1 levels were significantly higher in breast cancer samples than in paired non‐cancerous (normal) tissues (*P* < 0.0001; Figure 1A). In addition, this finding was supported by the observation that RRS1 mRNA levels were also higher in a panel of human breast cancer cell lines than in normal human mammary epithelial cells (HMECs) (*P* < 0.01, Figure 1B). Moreover, the corresponding RRS1 protein levels were assessed by Western blotting. RRS1 protein levels were increased in all three human breast cancer cell lines (*P* < 0.01, Figure 1C).

### 3.2 Correlations of RRS1 copy numbers and mRNA levels

To understand the association between the gene copy numbers and mRNA expression, the copy number variations (CNVs) of RRS1 was quantified by real‐time PCR in breast tumour tissues and paired tumour‐distant normal breast tissues from 24 breast cancer patients. RRS1 showed significant changes in CNVs in breast tumours, compared with those for normal breast tissues (T/N = 1.705, *P* = 0.0002) (Figure 1D). And the relative mRNA expression of RRS1 showed a significant correlation (*R* ^2^ = 0.438, *P* = 0.032) with its relative copy numbers.

### 3.3 RRS1 expression in breast tissues and its clinicopathological significance in breast cancer patients

**Figure 2:**
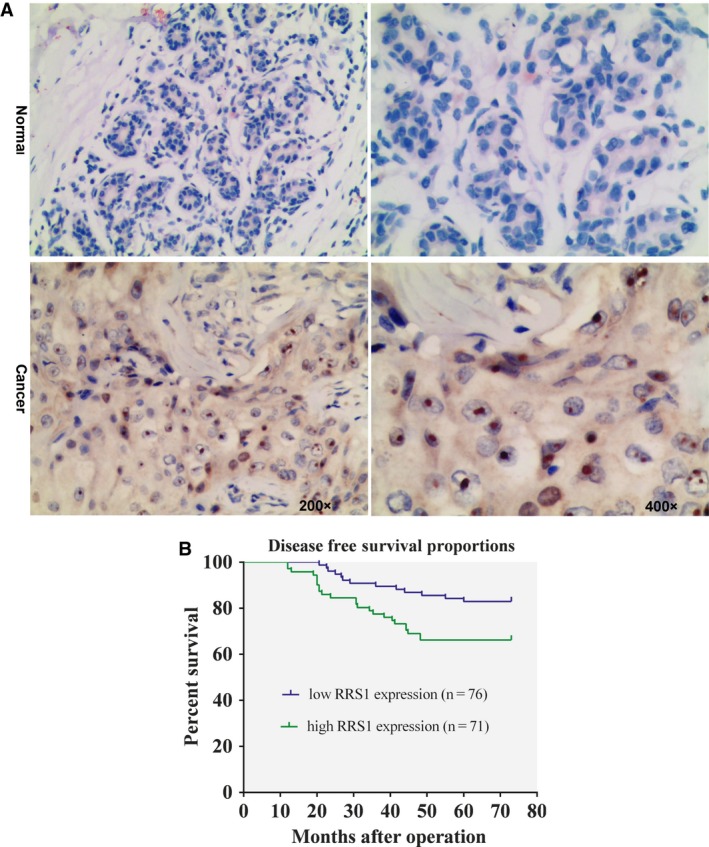
RRS1 overexpression in breast cancer correlated with lower disease‐free survival (DFS). A, RRS1 expression in primary breast cancer tissues and paired non‐cancerous (normal) tissues detected by IHC staining. B, Kaplan‐Meier survival curves for patients with breast cancer divided by RRS1 protein expression (*P* < 0.05)

Standard immunohistochemical analyses demonstrated that RRS1 was predominantly expressed in the nucleolus of breast tumour epithelial cells (Figure 2A), and 60.7% (147/242) of patients show overexpression of RRS1. When breast cancer patients were stratified by their RRS1 expression, the patients with high RRS1 expression levels exhibited lower DFS than patients with low levels of RRS1 (*P* < 0.05; Figure 2B). However, a Kaplan‐Meier analysis showed that there was no correlation between increased RRS1 levels and OS in patients with breast cancer (*P* = 0.063). In addition, we analysed the association between the clinicopathological variables and RRS1 expression in 242 breast cancer samples. As shown in Table 1, we found that RRS1 expression was significantly correlated with lymph node status, ER status, PR status, HER‐2 status and Ki67 proliferation index values according to chi‐square tests. However, RRS1 expression had no significant correlation with tumour size, age or TNM stage. These data indicate that RRS1 overexpression occurs during breast cancer progression.

**Table 1:** RRS1 Expression according to the clinicopathologic variables

Variables	Cases (n)	RRS1 expression		χ^2^	*P*
		High (n)	low (n)		
Age					
≤50 years	146	84	62	1.590	0.207
>50 years	96	63	33		
Tumour size					
≤20 mm	136	81	55	0.183	0.669
21‐50 mm	106	66	40		
Lymph node status					
Negative	146	80	66	5.463	0.019^a^
Positive	96	67	29		
TNM stage					
0/I	118	73	45	2.788	0.248
II	68	45	23		
III	56	29	27		
ER status					
Negative	107	47	60	22.753	0.000^a^
Positive	135	100	35		
PR status					
Negative	135	73	62	20.907	0.000^a^
Positive	107	74	33		
Her‐2 status					
Negative	169	115	54	12.532	0.000^a^
Positive	73	32	41		
Ki67 PI (%)					
≤10%	68	47	21	15.700	0.000^a^
11%‐32%	45	36	9		
≧33%	129	64	65		

Statistical analyses were performed by χ^2^ test.

a *P* < 0.05.

### 3.4 RRS1 knockdown suppresses proliferation and induces apoptosis in breast cancer cells

**Figure 3:**
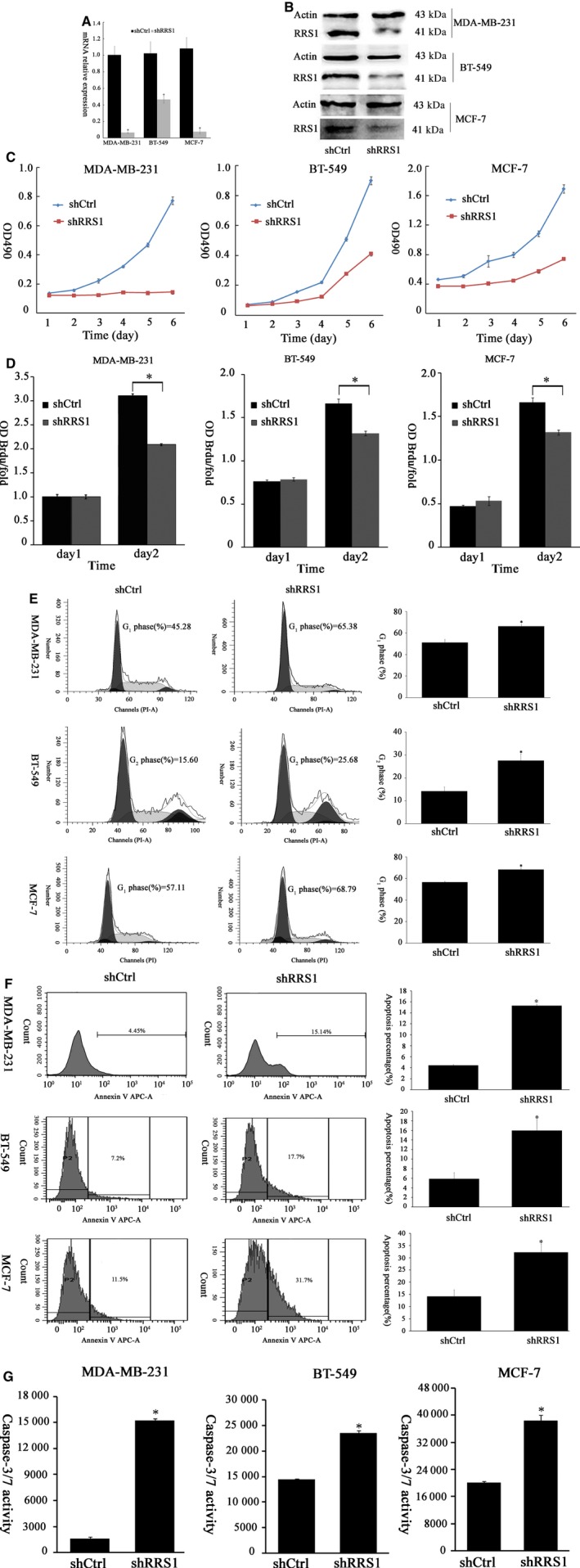
RRS1 knockdown inhibits proliferation and induces apoptosis in breast cancer cells. MDA‐MB‐231, BT‐549 and MCF‐7 cells were infected with a retrovirus expressing shRRS1 (shRRS1) or with a control vector (shctrl). A, RRS1 mRNA was measured (*P < 0.01 vs shctrl). B, RRS1 protein was detected. C, Cells were subjected to MTT assays at 24‐h intervals (for MDA‐MB‐231 cells at 2‐6 days, *P* < 0.001 for shctrl vs shRRS1; for BT‐549 cells at 3‐6 days, *P* < 0.001 for shctrl vs shRRS1; for MCF‐7 cells at 1‐6 days, *P* < 0.001 for shctrl vs shRRS1). D, Cells were subjected to BrdU incorporation assays (for MDA‐MB‐231 and BT‐549 cells at day 3, *P < 0.0001; for MCF‐7 cells at day 3, *P = 0.0005). E, The cell cycle was analysed by flow cytometry (there was a significant increase in the percentage of cells in the G1 phase for MCF‐7 and MDA‐MB‐231 cells, *P < 0.001 vs shctrl; BT‐549 cells displayed significant G2/M phase arrest, *P = 0.0024 vs shctrl). F, Flow cytometry was used to detect the percentage of Annexin V‐positive cells. (*P < 0.01, **P < 0.0001 vs shctrl). G, Caspase‐Glo3/7 assays were used to detect the activity of caspase‐3 and caspase‐7 (*P < 0.0001 vs shctrl)

Because RRS1 is highly overexpressed in breast cancer tissues, we investigated whether RRS1 depletion in human breast cancer cells would alter their proliferation ability. To test this hypothesis, we knocked down RRS1 in MDA‐MB‐231, BT‐549 and MCF‐7 cells. While these three cell lines overexpress RRS1, they represent distinct subgroups of breast cancer. In all three cell lines, RRS1 knockdown at both the mRNA and protein levels resulted in a significant reduction in cell proliferation (Figure 3A‐D). To investigate the molecular mechanism underlying the regulation of cell proliferation by RRS1, we cultured RRS1 knockdown MDA‐MB‐231, BT549 and MCF‐7 cells and evaluated their cell cycle distribution. Upon RRS1 knockdown, there was a 11%‐15% increase in G1 phase distribution of MCF‐7 and MDA‐MB‐231 cells, while the G2/M proportion of BT‐549 cells was significantly increased compared with the control‐infected cells (shCtrl) (Figure 3E). Apoptosis analysis was conducted by flow cytometry. The results demonstrated that there was a significant increase in the percentage of cells that were Annexin V‐positive in all three RRS1 knockdown cell lines (Figure 3F). Moreover, RRS1 knockdown significantly increased the activity of caspase‐3 and caspase‐7 (Figure 3G). Therefore, RRS1 knockdown in breast cancer cells induced apoptosis and caused detectable alterations in the cell cycle distribution.

### 3.5 RRS1 knockdown inhibits the formation and growth of murine xenografts

**Figure 4:**
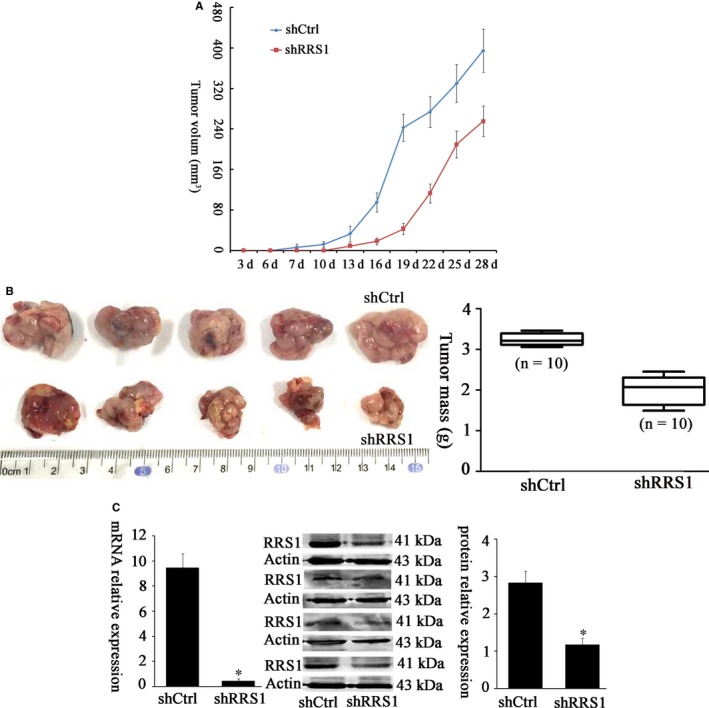
RRS1 knockdown inhibits breast cancer growth in vivo. MDA‐MB‐231 cells expressing RRS1‐targeting shRNA (shRRS1) or non‐targeting shRNA (shctrl) were subcutaneously injected into the flank of each mouse (n = 10 per group). A, Tumour volume was calculated at the indicated intervals (*P* < 0.001 for shctrl vs shRRS1 for days 13‐28). B, At the time of killing, the tumours were extracted, photographed and weighed (*P = 0.0001 vs shctrl). A representative photo of the two groups is shown. C, RNA and protein were extracted from the tumours, and RRS1 levels were detected (*P < 0.05 vs shctrl) (n = 10 per group)

To examine the role of RRS1 in the formation and development of mammary tumours in vivo, RRS1 knockdown MDA‐MB‐231 cells and control cells were subcutaneously injected into the flanks of athymic nude mice. Robust tumours developed from the control MDA‐MB‐231 cells within 28 days. In contrast, tumour growth was significantly lower in mice injected with shRRS1 MDA‐MB‐231 cells (Figure 4A, *P* < 0.05). At the time of killing (day 28 after treatment), tumours derived from shRRS1 MDA‐MB‐231 cells were smaller in volume (255.29 vs 394.62 mm^3^; *P* = 0.0122) and weighed significantly less than the tumours from control mice (Figure 4B, *P* = 0.0001). These tumours also showed reduced RRS1 protein and mRNA levels (Figure 4C, *P* < 0.05), indicating that RRS1 shRNA expression persisted over the course of the experiment.

### 3.6 RRS1 knockdown activated p53 and p21

**Figure 5:**
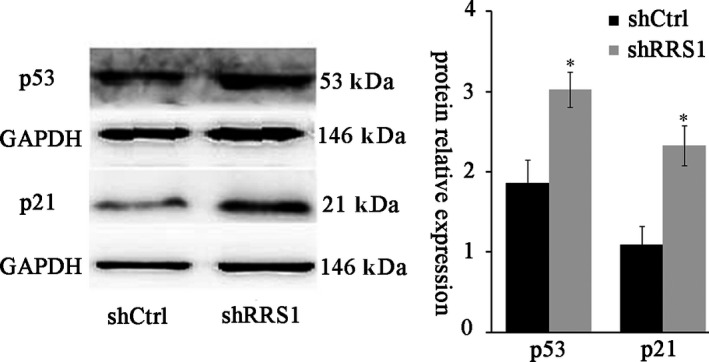
P53 and p21 are induced by RRS1 knockdown. MCF‐7 cells were infected with a retrovirus expressing RRS1 (shRRS1) or with a Ctrl vector (shctrl) for 2 days. Whole‐cell lysates were analysed by Western blot. P53 and p21 expression levels were increased by RRS1 knockdown (*P < 0.05 vs shctrl)

A previous study revealed that the nucleolus senses various stresses and plays a co‐ordinating role in activating p53.^31^ The mechanisms underlying how RRS1 knockdown inhibits proliferation in breast cancer are currently unknown; however, because RRS1 is one of the rRNA processing factors in ribosome biogenesis, we speculated that it may affect breast cancer proliferation by activating p53. We knocked down RRS1 in MCF‐7 cells and investigated whether RRS1 knockdown activates p53 and its downstream target p21. Our results revealed that RRS1 knockdown increased the levels of p53 and its downstream target p21 and induced cell cycle arrest (Figure 5).

### 3.7 RPL11 and MDM2 are involved in the RRS1 knockdown‐mediated growth inhibition of breast cancer cells

**Figure 6:**
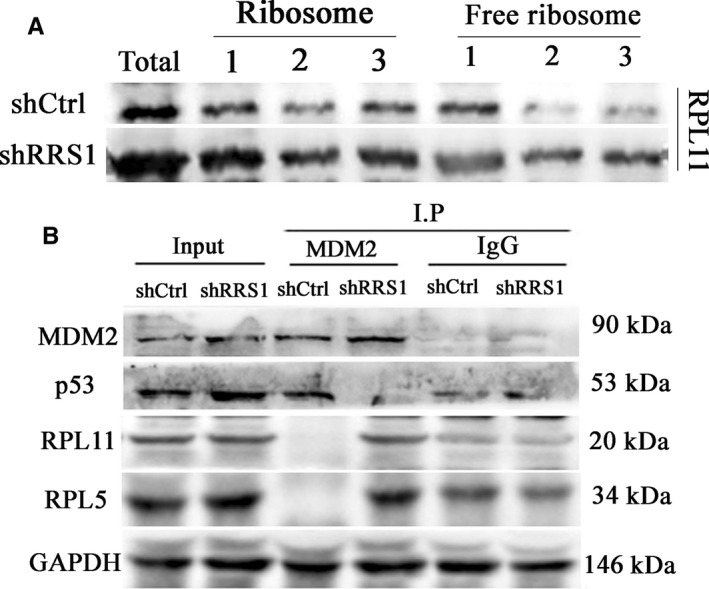
RPL11/RPL5 and MDM2 are involved in RRS1‐mediated growth inhibition. MCF‐7 cells were infected with a retrovirus expressing RRS1 (shRRS1) or with a Ctrl vector (shctrl) for 2 days. A, Ribosomal (Ribo) and non‐ribosomal (Non‐Ribo) fractions were isolated by sucrose gradient centrifugation and immunoblotted using an RPL11/RPL5 antibody. B, Cell lysates were immunoprecipitated with an anti‐MDM2 antibody and were immunoblotted with the indicated antibody

A previous study showed that a common feature of RRS1 knockdown is an increase in the nucleoplasmic accumulation of RPL11.^21^ We predicted that ribosome‐free RPL11 and MDM2 would be involved in breast cancer proliferation inhibition upon RRS1 knockdown. Thus, we evaluated the levels of ribosome‐free RPL11 in MCF‐7 cells by isolating ribosomal (Ribo) and non‐ribosomal (Non‐Ribo) fractions from RRS1 knockdown cells and control cells. Western blot analyses showed that the levels of ribosomal RPL11 decreased significantly in shRRS1 cells, while non‐ribosomal RPL11 increased correspondingly (Figure 6A). In addition, we also measured the levels of MDM2, which interacts with p53 and RPL11/RPL5. The results of co‐immunoprecipitation experiments showed that compared with the control, RRS1 knockdown reduced the interaction between MDM2 and p53 (Figure 6B). In contrast, the interaction between MDM2 and RPL11/RPL5 was enhanced (Figure 6B). These results indicate that RRS1 knockdown increases the levels of non‐ribosomal RPL11, which binds to and inhibits MDM2 to activate p53.

## 4 DISCUSSION

Ribosome protein expression is commonly misregulated in cancer.^32^, ^33^ In this study, we described for the first time that RRS1 is overexpressed in breast cancer. By analysing breast tissues, we observed that RRS1 mRNA was expressed at higher levels in breast cancer tissues than in paired non‐cancerous tissues. And the relative mRNA expression of RRS1 showed a significant correlation with its relative copy numbers. RRS1 overexpression occurred in 60.7% of the tumours and significantly correlated with DFS in breast cancer patients. Our data also showed a correlation between RRS1 expression and ER status, PR status, Ki67 proliferation index values and lymph node status. Moreover, an in vitro analysis indicated that RRS1 mRNA and protein levels were significantly increased in a panel of human breast cancer cell lines. These results support further investigation of RRS1 as a promising therapeutic target for breast cancer.

We have demonstrated here that RRS1 plays a functional role in breast cancer cell proliferation. Experimentally, RRS1 knockdown in breast cancer cells significantly reduced cell proliferation and tumour development in a mouse xenograft model. It has been reported that RRS1 contributes to chromosome congression and results in mitotic delay in human HeLa cells, suggesting that RRS1 is essential for cell cycle progression.^34^ Our results indicated that RRS1 knockdown induced distinct cell cycle arrest. Furthermore, RRS1 knockdown induced cell apoptosis. In addition to our results, the functional role of RRS1 in human colorectal and hepatocellular carcinomas has also been reported.^19^, ^20^ Both papers conclude that RRS1 plays important roles in the proliferation of human cancers.

However, it is important to understand how RRS1 regulates cell proliferation in breast cancer. The tumour suppressor p53 acts as an important regulator of cellular stresses by inducing distinct classes of genes involved in cell senescence, apoptosis, cell cycle arrest and DNA repair.^35^, ^36^, ^37^ Our results found that RRS1 knockdown resulted in the accumulation of p53 and p21 in breast cancer cells. However, how RRS1 activates p53 remains unclear. Interestingly, the depletion of RRS1 resulted in an increase in the nucleoplasmic accumulation of both RPL5 and RPL11.^21^ RPL5 and RPL11 activate p53 by down‐regulating MDM2 in the nucleoplasm.^38^ MDM2, an E3 ubiquitin, inhibits the activity of p53 through proteasome‐mediated degradation.^39^ The results of this study indicated that RRS1 knockdown caused a marked increase in ribosome‐free RPL11 levels and a consequential reduction in ribosomal RPL11 levels in breast cancer cells. In addition, our CoIP experiments also confirmed that MDM2 induced by RRS1 knockdown was in direct contact with RPL11 and p53. In fact, we should further explore the other two components of 5SRNP, RPL5 and 5sRNA, which are critical for p53 activation and cell growth, upon RRS1 knockdown in breast cancer.

## 5 CONCLUSION

In this study, we demonstrate for the first time that the RRS1 and RRS1/RPL11/p53 signal axes are involved in breast cancer proliferation. High RRS1 expression levels were associated with poor breast cancer prognosis, and RRS1 knockdown inhibited breast cancer proliferation in vitro and in vivo. These findings strongly suggest that RRS1 may contribute to breast cancer growth and survival. RRS1 may be characterized as a biomarker and could provide a new possible target for breast cancer treatment.

## CONFLICT OF INTEREST

The authors declare that they have no competing interests.
